# Evaluation of Swallowing and Tongue Strength Index in Patients With Temporomandibular Dysfunction: A Cross‐Sectional Study

**DOI:** 10.1155/ijod/2351314

**Published:** 2026-04-28

**Authors:** Julia da Silva Germiniani, Milena Sampaio Kuczera, Aline Xavier Ferraz, Rosane Sampaio Santos, Bianca Simone Zeigelboim, Cristiano Miranda de Araujo, Glória Maria Nogueira Cortz Ravazzi, Flavio Magno Gonçalves, José Stechman-Neto

**Affiliations:** ^1^ Postgraduate Program in Human Communication Health, Tuiuti University of Paraná, Curitiba, Paraná, Brazil; ^2^ Postgraduate Program in Dentistry, Tuiuti University of Paraná, Curitiba, Paraná, Brazil

**Keywords:** chewing muscles, deglutition, stomatognathic system, tongue strength

## Abstract

Temporomandibular disorders (TMDs) can affect the stomatognathic system (SS) and alter tongue performance. This cross‐sectional study aimed to evaluate tongue strength and its relationship with swallowing function in individuals diagnosed with TMD. Fifty adults participated (32 with TMD and 18 asymptomatic controls). TMD diagnosis was established according to the Diagnostic Criteria for TMDs (DC/TMD). Swallowing function was screened using the Eating Assessment Tool (EAT‐10), and tongue strength was assessed using the Pro‐Fono Lip and Tongue Pressure (PLTP) Biofeedback device. The sample had a mean age of 37.7 years and included 12 males and 38 females. Within the TMD group, 11 participants were diagnosed with myalgia, 8 with arthralgia, and 13 with both conditions. According to the EAT‐10, 40 participants scored below 3, whereas 10 scored 3 or higher, indicating potential swallowing risk. Individuals with TMD exhibited significantly lower tongue strength in both the dorsum and tip compared with controls (*p* < 0.05). No significant differences were observed between sexes or age groups, and no associations were found between tongue strength and EAT‐10 scores. Although self‐perceived swallowing difficulties did not differ between groups, reduced tongue strength was observed in individuals with TMD, highlighting the importance of including objective tongue strength assessment in the clinical evaluation and management of these patients.

## 1. Introduction

Temporomandibular disorders (TMDs) comprise a group of painful and/or dysfunctional conditions affecting the masticatory muscles, the temporomandibular joints (TMJs), and related structures [1]. The symptoms most frequently reported by affected individuals include myalgia, arthralgia, limited mandibular range of motion, and joint sounds [2].

Epidemiological studies indicate that more than 50% of the population may present at least one sign or symptom of TMD during their lifetime [3–5]. However, most affected individuals do not require treatment, with estimates suggesting that only a small proportion seek clinical intervention, ranging from 3.6% to 7% [6–8].

The impacts of TMD on the motor functions of the orofacial region have been extensively investigated; nevertheless, important gaps remain regarding specific targets for functional assessment and rehabilitation [1]. This includes the analysis of tongue strength and its correlation with swallowing function, which remains insufficiently explored [9]. The TMJs and the tongue are part of the stomatognathic system (SS) and must function in a coordinated manner to ensure the efficient execution of orofacial tasks, including swallowing [10, 11].

Studies have demonstrated a significant relationship between the presence of TMD and altered swallowing patterns [12]. Abnormal swallowing patterns were observed in up to 76% of patients with TMD, suggesting that these individuals may adopt adaptive strategies to avoid pain [13]. A relationship between facial pain and abnormal swallowing has been identified, which has been attributed, at least in part, to increased activity of the digastric and other suprahyoid muscles [13].

Although TMDs are highly prevalent and well‐characterized in terms of pain and joint dysfunction, the combined assessment of tongue strength and swallowing function remains underexplored. Understanding how neuromuscular adaptations associated with these findings suggest a possible relationship and swallowing efficiency is clinically relevant for both diagnosis and rehabilitation. Recent studies have emphasized the need for standardized protocols and objective measurements to clarify these associations and to guide targeted therapies [14, 15].

The null hypothesis of this study was that there are no significant differences in tongue strength between individuals with TMDs and asymptomatic controls, and that no significant bivariate associations would be observed between tongue strength and age, sex, body mass index (BMI), or self‐reported swallowing difficulties (as assessed by the Eating Assessment Tool‐10 [EAT‐10]).

Therefore, the primary objective of this study was to determine whether individuals with TMD exhibit reduced tongue strength compared to asymptomatic controls. Additionally, secondary exploratory objectives included exploring potential bivariate associations between tongue strength indices and sex, age, BMI, and self‐reported swallowing symptoms, and self‐reported swallowing symptoms on tongue strength indices. Given the limited evidence available, this study adopts an exploratory cross‐sectional design to generate preliminary evidence regarding the relationship between TMD and tongue strength.

## 2. Methodology

### 2.1. Study Design and Ethical Considerations

This was an observational, cross‐sectional, and quantitative study conducted with a convenience sample. The research protocol was approved by the Research Ethics Committee of Universidade Tuiuti do Paraná (Approval Number: 68579323.7.0000.8040). All participants were informed about the study objectives and procedures and signed the Informed Consent Form prior to enrollment.

### 2.2. Study Setting and Period

Data collection was carried out between April and October 2023 at the Centro de Diagnóstico das Alterações Temporomandibulares (CDATM) and the Speech‐Language Pathology Clinic (Swallowing Laboratory) of Universidade Tuiuti do Paraná, Curitiba, Brazil. Participants were recruited on a voluntary basis among patients seeking specialized care at these facilities.

### 2.3. Examiner Calibration

Before data collection, the research team underwent calibration to standardize all examination and data collection procedures. Both theoretical instruction and practical training were conducted to ensure the reliability and reproducibility of measurements, thereby minimizing potential observer bias. Interrater agreement was considered excellent, with intraclass correlation coefficients (ICCs) exceeding 0.80.

### 2.4. Participants

The sample included adult individuals of both sexes who answered “yes” to question 4D of the Diagnostic Criteria for TMDs (DC/TMDs) questionnaire and reported swallowing‐related symptoms assessed using the EAT‐10.

Exclusion criteria included pediatric patients, syndromic individuals, and those with a history of head and neck cancer, neurological disorders, prior orofacial surgery, or concurrent treatment for conditions that could interfere with the study outcomes. During the recruitment period, all individuals who met the eligibility criteria and agreed to participate were included in the study. No participants were excluded after enrollment. All collected data were complete, and no missing data were identified for the primary variables analyzed. Therefore, no imputation procedures were required.

Given the small and convenience‐based sample, a participant flow diagram was not constructed.

### 2.5. Procedures and Instruments

#### 2.5.1. Clinical Evaluation—DC/TMD

All participants were evaluated using the DC/TMD, with the diagnosis clinically confirmed by an experienced specialist. This protocol, routinely applied at the CDATM, enabled the identification of signs and symptoms of TMDs, encompassing both physical and psychosocial dimensions.

#### 2.5.2. Swallowing Assessment—EAT‐10

Swallowing function was assessed using the EAT‐10, a self‐administered questionnaire consisting of 10 items, with a total score ranging from 0 to 40. Scores equal to or greater than three indicated an increased risk for dysphagia. The questionnaire was completed individually by each participant via an online Google Forms platform.

#### 2.5.3. Tongue Strength Assessment

Tongue strength was measured using the Pro‐Fono Lip and Tongue Pressure (PLTP) Biofeedback device. This system includes a pressure sensor connected to an air‐filled bulb, recording data in kilopascals (kPa) through a graphical output generated by the device software. Prior to data collection, the device underwent calibration according to the manufacturer’s recommendations to ensure measurement accuracy. Measurement error was monitored throughout testing and remained within the acceptable range specified by the manufacturer. Additionally, repeatability tests were conducted during the training phase, yielding ICCs greater than 0.80, indicating adequate procedural reliability.

**Figure 1:**
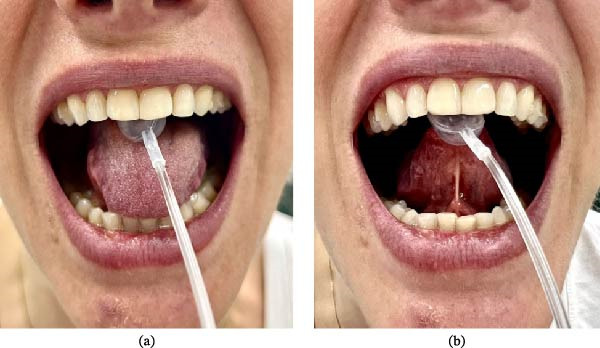
Standardized procedures for tongue pressure assessment using the PLTP device: (a) measurement of tongue dorsum pressure against the palate; (b) measurement of tongue tip pressure against the palate. *Source:* Author’s own.

**Figure 2:**
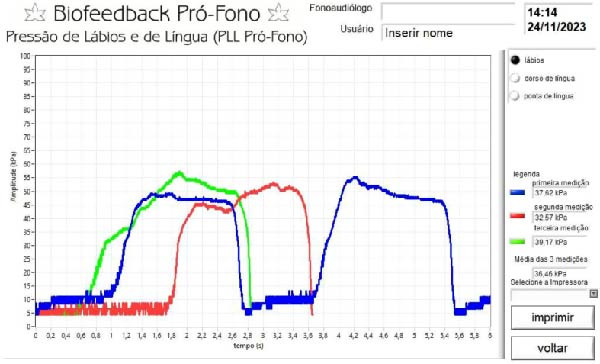
Example of the graphical output generated by the device software after tongue pressure measurement, showing the pressure curve recorded during the task. *Source*: Author’s own.

The test was performed in two stages: (1) tongue dorsum pressure and (2) tongue tip pressure (Figure 1). Each stage consisted of three 5‐s trials, interspersed with 40‐s rest intervals. The mean value of the three measurements was automatically calculated by the software (Figure 2). During testing, participants remained seated with an upright posture, feet on the floor, and head aligned with the horizontal plane. Sex, height, and weight were recorded for BMI calculation.

#### 2.5.4. Variables Analyzed

Primary variables included the presence of TMDs (classified according to DC/TMD), EAT‐10 score, and tongue strength indices (dorsal and tip; in kPa). Secondary variables were age, sex, and BMI.

#### 2.5.5. Sample Size

No a priori sample size calculation was performed, as this study was based on a convenience sample composed of individuals who spontaneously sought evaluation and treatment at the university clinics during the study period. The final sample consisted of 50 participants who met the eligibility criteria and agreed to participate voluntarily.

#### 2.5.6. Statistical Analysis

Statistical analyses were conducted using nonparametric methods due to the distribution of the variables, as assessed by the Shapiro–Wilk test. Group comparisons between individuals with and without TMD were performed using the Mann–Whitney *U* test. Effect sizes were calculated using the *r* statistic, derived from the standardized *Z* value (*r* = *Z*/√*N*), as an approximation of the rank‐biserial correlation to estimate the magnitude of group differences, and were interpreted according to Cohen’s conventional thresholds [16], where *r* = 0.1 indicates a small effect, *r* = 0.3 a medium effect, and *r* ≥ 0.5 a large effect, acknowledging that these benchmarks are approximate when applied to nonparametric effect size estimates. Ninety‐five percent confidence intervals (95% CI) for the effect sizes were estimated using bootstrap resampling with 5000 iterations. Associations between tongue strength indices and continuous variables (EAT‐10 scores and BMI) were evaluated using Spearman’s rank correlation coefficient. Comparisons according to sex and age group were also conducted using bivariate nonparametric tests. A significance level of 5% (*p* < 0.05) was adopted for all analyses.

The analytical strategy was exploratory and based on independent bivariate comparisons. No hierarchical testing framework was applied, and no multivariable regression models or statistical adjustments for potential confounding variables were performed. Consequently, the analyses do not allow for the identification of independent associations. Additionally, no adjustment for multiple comparisons was performed, which increases the risk of type I error. Therefore, the findings should be interpreted with caution and considered hypothesis‐generating.

## 3. Results

The sample consisted of 50 participants, with a mean age of 37.7 ± 15.9 years. Of these, 12 were male (mean age: 41.1 ± 17.6 years) and 38 were female (mean age: 35.3 ± 15.3 years). Among all participants, 32 were diagnosed with TMD, while 18 were asymptomatic. Within the TMD group, 11 participants were diagnosed with myalgia, 8 with arthralgia, and 13 with both conditions.

Regarding the EAT‐10 results, 40 (80%) participants scored below 3, whereas 10 (20%) scored 3 or higher, suggesting a potential risk for swallowing alterations.

When comparing participants with and without TMD, a statistically significant difference was observed in tongue strength for both the tongue dorsum (*p* = 0.012) and the tongue tip (*p* = 0.009), indicating small and moderate effect sizes, respectively. Participants with TMD exhibited lower tongue strength in both regions (Table 1). Post hoc power analysis indicated a power of 0.65 for tongue dorsum strength, suggesting limited sensitivity to detect group differences, whereas tongue tip strength demonstrated a power of 0.81, indicating adequate statistical power. The effect sizes and their corresponding 95% CI are presented in Table 1.

**Table 1:** Comparison of median (IQR) tongue strength (kPa) between participants with and without TMD using the Mann–Whitney *U* test, including effect size (*r*) and 95% confidence intervals.

Variable	TMD group (*N* = 32)	Non‐TMD group (*N* = 18)	*p*‐Value ^∗^	Effect size (r) [ci95%]
Tongue dorsum strength (kPa)	39.28 (25.96–55.44)	61.87 (34.78–90.43)	0.046 ^∗^	0.28 [0.02–0.54]
Tongue tip strength (kPa)	41.01 (24.40–61.23)	61.81 (37.65–89.59)	0.009 ^∗^	0.37 [0.11–0.59]

*Note:* Effect size calculated using an approximation of the rank‐biserial correlation. Significance level set at 5%.  ^∗^Mann–Whitney *U* test.

Abbreviation: IQR, interquartile range.

No significant differences were observed in tongue strength between age groups (adults vs. older adults) or between sexes; however, sex‐related comparisons should be interpreted with caution due to the unequal sex distribution in the sample (38 women and 12 men). Likewise, no significant association was found between EAT‐10 scores and tongue strength indices. Spearman’s rank correlation analysis showed no significant associations between tongue strength (tongue dorsum or tongue tip), EAT‐10 scores, and BMI (*p* > 0.05 for all comparisons).

## 4. Discussion

In the present study, the null hypothesis was not supported, as individuals with TMDs exhibited lower tongue strength compared with asymptomatic controls. The magnitude of these differences was small for tongue dorsum strength and moderate for tongue tip strength. Lower tongue strength was consistently observed among individuals with TMD, suggesting differences in lingual muscle performance. The reported effect sizes, together with their 95% CI, provide an estimate of both magnitude and precision; however, the relatively wide intervals, particularly for tongue dorsum strength, indicate some degree of uncertainty. Although these impairments were not pronounced, the observed effect sizes suggest a potential pattern of difference.

An additional aspect that warrants consideration is the substantial variability observed within both groups, as reflected by the dispersion measures presented in the tables. This intragroup variability may indicate heterogeneity in clinical presentation, pain severity, functional adaptation, or unmeasured behavioral factors. Such heterogeneity may have influenced the magnitude and stability of the observed differences, potentially attenuating or amplifying group contrasts. Given the exploratory design and the absence of stratified or multivariable analyses, the impact of this variability cannot be fully determined. Future studies with larger samples and more refined subgroup characterization may help clarify whether distinct clinical phenotypes of TMD are differentially associated with tongue strength.

Rather than representing isolated muscular weakness, reduced tongue strength in individuals with TMD may reflect broader neuromuscular adaptations potentially associated with persistent orofacial pain. TMDs are multifactorial conditions that may present as acute or chronic and affect the TMJ and adjacent structures, particularly the masticatory muscles [17]. Chronic pain has been reported to influence motor control strategies, often leading to adaptive patterns aimed at minimizing discomfort while preserving function. In this context, pain‐related limitations in mandibular movement could potentially alter tongue activation and coordination during oral tasks, including swallowing [1, 9].

Although muscular adaptations during swallowing have been frequently reported in individuals with TMD, their impact on objective measures of tongue strength remains controversial. Previous studies have reported that abnormal swallowing patterns may be present in up to 76% of patients with TMD, possibly reflecting compensatory strategies to avoid pain [12, 13]. These adaptations may allow the maintenance of functional swallowing despite measurable reductions in muscle strength, which is consistent with the small‐to‐moderate effect sizes observed in the present study, although this interpretation remains speculative.

The tongue plays a central role in the SS and is essential for bolus propulsion and pressure generation against the palate [18–20]. Impairments in tongue pressure have been associated in previous investigations with pharyngeal residue and swallowing disorders [21, 22]. Tongue pressure is therefore considered an important indicator of orofacial motor performance, although it represents only one dimension of functional capacity [23]. In the present study, individuals with TMD demonstrated reduced strength in the tongue dorsum and tip, which aligns with previous findings suggesting compromised lingual performance in this population [9, 14, 24–26]. However, the mechanisms linking TMD to reduced tongue strength remain hypothetical and warrant further investigation using neurophysiological and functional assessments.

The absence of significant associations between tongue strength and self‐reported swallowing symptoms, as measured by the EAT‐10, provides additional insight into these findings. Although the EAT‐10 is a widely used screening tool for swallowing risk [27], and scores equal to or greater than 3 indicate increased risk [28, 29], most participants in the present study scored below this threshold. No statistically significant differences in tongue strength were observed between individuals with lower and higher EAT‐10 scores. This finding may suggest that subjective swallowing complaints do not necessarily capture subtle neuromuscular impairments detected through objective strength measurements. The relationship between perceived swallowing difficulty and tongue strength remains incompletely established and may be influenced by compensatory mechanisms and individual pain adaptation.

Age, sex, and BMI were not statistically significantly associated with tongue strength in the present sample. Although age‐related declines in tongue strength have been reported, these changes tend to occur gradually and are more evident in older populations [30]. The relatively young mean age of the participants may partly explain the absence of detectable age‐related effects. Regarding sex, although male participants showed higher average tongue strength values, comparisons should be interpreted cautiously due to the unequal sex distribution in the sample, which may have limited statistical power. Previous studies suggest that males generally exhibit greater tongue pressure and resistance, although age‐related declines have also been reported [30]. With respect to BMI, the lack of statistically significant associations is consistent with the divergent findings reported in the literature and highlights the need for further investigation into the influence of anthropometric factors on tongue strength [15].

Overall, the present findings are consistent with previous evidence indicating reduced tongue strength in individuals with TMD [9, 14, 15, 18], while also suggesting that these impairments may be small to moderate in magnitude and not necessarily accompanied by self‐perceived swallowing dysfunction. Pain‐related inhibition, altered mandibular kinematics, and changes in tongue resting posture have been proposed as potential mechanisms underlying these alterations; however, these pathways remain theoretical. These factors may act synergistically, potentially resulting in measurable reductions in strength without overt functional failure.

Several limitations should be acknowledged. Tongue strength assessment was limited to the tongue dorsum and tongue tip, while other relevant dimensions such as endurance, protrusion strength, and pressure during swallowing were not evaluated. The relatively small sample size and cross‐sectional design limit generalizability and preclude causal inferences. Additionally, the lack of standardized and validated protocols for maximum tongue and lip strength assessment hampers comparability across studies. Swallowing was assessed exclusively through a screening questionnaire, which does not replace instrumental evaluations such as videofluoroscopy. Important potential confounding variables were not analytically controlled. Although demographic and clinical variables were explored separately, no multivariable modeling was performed to determine independent effects. This limits the ability to attribute the observed differences specifically to TMD. Future research should include larger and more diverse samples, investigate additional dimensions of tongue function, and incorporate neurophysiological and instrumental swallowing assessments to better elucidate the mechanisms underlying tongue strength reductions in individuals with TMD. Longitudinal studies and randomized clinical trials would be necessary to explore potential causal relationships and to evaluate interventions targeting lingual strength and orofacial function in this population. The modest sample size, selected by convenience, and the absence of an a priori power calculation limit the inferential strength and external validity of the findings.

Taken together, the methodological decisions adopted in this study should be interpreted within the context of an exploratory cross‐sectional design. Multiple independent comparisons, including subgroup and secondary analyses, were conducted without hierarchical structuring or adjustment for multiplicity, which increases the risk of type I error and the identification of spurious associations. Therefore, the findings should be interpreted with caution and considered hypothesis‐generating rather than confirmatory.

## 5. Conclusion

Based on the findings of this exploratory study, lower tongue strength values were observed in individuals with TMDs compared with asymptomatic individuals, particularly in the anterior region of the tongue. No significant differences were identified between groups regarding self‐perceived swallowing symptoms. These results should be interpreted with caution, considering the study’s limitations, including the small and unbalanced sample size, the cross‐sectional design, and the absence of instrumental swallowing assessment. Within these limitations, the findings may suggest a possible association between TMD and reduced tongue strength, without corresponding differences in swallowing self‐perception. Further studies with larger samples, longitudinal designs, and comprehensive swallowing assessments are necessary to better understand the relationship between TMD, lingual function, and swallowing outcomes.

## Author Contributions

Julia da Silva Germiniani contributed to conception, data collection, design, drafting, and critical review of the manuscript. Milena Sampaio Kuczera contributed to conception, data collection, design, and critical review of the manuscript. Aline Xavier Ferraz contributed to critical review of the manuscript. Flavio Magno Gonçalves contributed to conception, design, statistical analysis, and critical review of the manuscript. Glória Maria Nogueira Cortz Ravazzi contributed to critical review of the manuscript. Bianca Simone Zeigelboim contributed to critical review of the content. Cristiano Miranda de Araujo contributed to statistical analysis of the manuscript. Rosane Sampaio Santos contributed to critical review of the manuscript. José Stechman‐Neto supervised, contributed to data interpretation, and critical review of the manuscript.

## Funding

This study was financed in part by the Coordenação de Aperfeiçoamento de Pessoal de Nível Superior—Brazil (CAPES)—Finance Code 88887.847808/2023‐00.

## Disclosure

All authors gave final approval and agreed to be accountable for all aspects of the work.

## Conflicts of Interest

The authors declare no conflicts of interest.

## Data Availability

The data that support the findings of this study are available upon request from the corresponding author. The data are not publicly available due to privacy or ethical restrictions.
